# Vaccine hesitancy and (fake) news: Quasi‐experimental evidence from Italy

**DOI:** 10.1002/hec.3937

**Published:** 2019-08-20

**Authors:** Vincenzo Carrieri, Leonardo Madio, Francesco Principe

**Affiliations:** ^1^ Department of Law, Economics and Sociology “Magna Graecia” University Catanzaro Italy; ^2^ RWI Research Network Essen Germany; ^3^ HEDG University of York York UK; ^4^ CORE Université Catholique de Louvain Louvain‐la‐Neuve Belgium; ^5^ CESifo Research Network Munich Germany; ^6^ Erasmus School of Economics Erasmus University Rotterdam Rotterdam The Netherlands

**Keywords:** child immunization rates, fake news, Internet, social media, vaccine hesitancy

## Abstract

The spread of fake news and misinformation on social media is blamed as a primary cause of vaccine hesitancy, which is one of the major threats to global health, according to the World Health Organization. This paper studies the effect of the diffusion of misinformation on immunization rates in Italy by exploiting a quasi‐experiment that occurred in 2012, when the Court of Rimini officially recognized a causal link between the measles‐mumps‐rubella vaccine and autism and awarded injury compensation. To this end, we exploit the virality of misinformation following the 2012 Italian court's ruling, along with the intensity of exposure to nontraditional media driven by regional infrastructural differences in Internet broadband coverage. Using a Difference‐in‐Differences regression on regional panel data, we show that the spread of this news resulted in a decrease in child immunization rates for all types of vaccines.

## 1 INTRODUCTION

Several countries are experiencing outbreaks of vaccine‐preventable diseases, such as measles and diphtheria. For example, in 2018, measles cases increased by 30% globally (World Health Organization, 2019). On 29 January 2019, Washington State officially declared a state of emergency due to a measles epidemic. In Europe, between 1 February 2017, and 31 January 2018, the European Surveillance System reported 14,732 cases of measles. Among European countries, Italy (4,978 cases) had the highest incidence, just after Romania (5,224 cases; European Centre for Disease Prevention and Control, 2019). These worrying statistics led the World Health Organization to include Vaccine hesitancy—that is, the reluctance or refusal to vaccinate despite the availability of vaccines—as one of top 10 threats to global health today.

The spread of fake news and misinformation on social media is blamed as a primary cause of vaccine hesitancy (Aquino et al., 2017; Dube, Vivion, & MacDonald, 2015; Jolley & Douglas, 2014; Smith & Marshall, 2010). This originated from the measles‐mumps‐rubella (MMR)—autism controversy that stemmed from Andrew Wakefield's fake study. A number of papers found that this controversy had a significant effect on immunization choices. Anderberg, Chevalier, and Wadsworth (2011) found an effect on the uptake of the MMR vaccine in the United Kingdom, which dropped by over 5% points in 5 years, before rising again. Similarly, Smith, Ellenberg, Bell, and Rubin (2008) examined MMR uptake and nonreceipt in the United States and found declines in 1999 and 2000 and then a return to previous levels of vaccination. More recently, Chang (2018) showed that controversy led to a decline in MMR immunization rates and negative spillovers onto the use of other vaccines in the United States.

**Figure 1:**
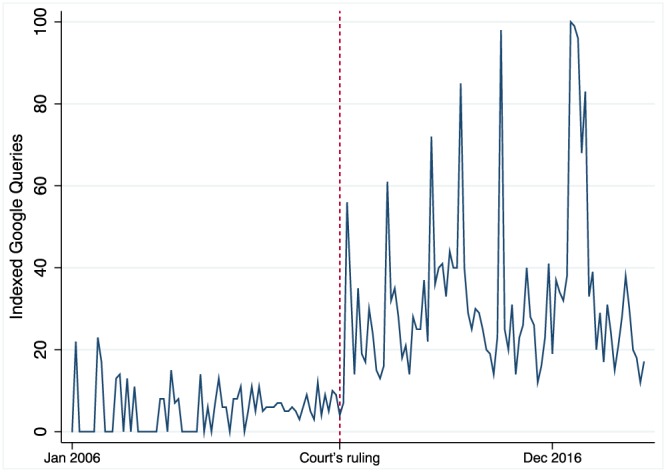
Google trends for “vaccini autismo” (vaccines autism) in Italy, 2006–2018. Own elaboration on Google Trends data [Colour figure can be viewed at http://wileyonlinelibrary.com]

This paper complements these existing studies in two important ways. First, it exploits a quasi‐experiment that occurred in March 2012 in Italy, when the Court of Rimini granted compensation to a family after recognizing that the MMR vaccine caused their child's autism. To our knowledge, this was the first time that an official body formally recognized a causal link between the MMR vaccine and autism. The decision was initially covered by the most read national media outlets (e.g., La Repubblica and Il Corriere della Sera). However, following the court's decision, people's concerns about vaccine side effects were proliferated on the Internet. Misinformation and fake news surrounding vaccines, now supported by a judge, went viral. Figure 1 shows that the number of queries for “vaccines and autism” on the search engine Google increased drastically after March 2012 and remained quite stable afterwards. Compared with pre‐2012, the volume of searches increased by 600%. Indeed, as also supported by the relevant medical literature such as Aquino et al. (2017) and Donzelli et al. (2018), the court's ruling allows us to establish a crucial trigger for the virality of (fake) news and misinformation surrounding vaccines in Italy.

Second, as access to nontraditional media and exposure to misinformation is facilitated by Internet availability, we exploit the heterogeneity in regional (NUTS‐2 level) broadband coverage across areas of the country. Broadband coverage depends on the local historical infrastructural system, which has undergone several structural changes in the period we considered, to bridge the long‐lasting “Digital Divide” in Italy. In the period analyzed, broadband coverage in Italy passed from 15% in 2006 to 76% in 2016. This was essentially due to the “Digital Italy” plan launched by the Italian Government in 2008 to reach the ambitious goals of “Europe 2020,” the strategy of the European Union to ensure full broadband access (up to 30 Mbps) for all Europeans by 2013 and 50% ultra‐broadband coverage (up to 100 Mbps) by 2020. Basically, all territorial areas were supposed to reach these goals and, with different intensities and timings, were exposed to broadband deployment and upgrade. In practice, the rate of change in broadband coverage was dependent on the historical condition of the telephone line network. This is because the broadband network basically exploits the regular copper phone lines once adapted with xDSL technologies (Infratel, 2011). Further, the complex orography of the territory makes the adaptation of the phone lines even more difficult in some areas, and this represents a further source of heterogeneity in broadband coverage across the Italian territory. As a result, the changes in broadband coverage are thus unlikely to be correlated with the demand for high‐speed Internet, and this provides an exogenous variation in the regional exposure to news. Similar identification strategies using discontinuities in broadband coverage have been widely used to estimate the effect of the Internet and media exposure on other relevant outcomes (see, e.g., Falck, Gold, & Heblich, 2014; Gavazza, Nardotto, & Valletti, 2018).

We combine both sources of variations (i.e., the 2012 court ruling and heterogeneity in broadband coverage) in a Difference‐in‐Differences (DiD) framework. We find that the spread of misinformation surrounding vaccines following the court's ruling caused a significant reduction in child immunization rates.

## 2 METHODS AND DATA

We use a unique longitudinal dataset recording regional data on child immunization rates in Italy, matched with information on broadband coverage from 2006 to 2016 for all 21 NUTS‐2 areas (19 regions and two autonomous provinces). 1In Italy, immunization programs are managed in the context of the National Health Service, which provides universal health coverage by setting the core benefits package of health services to be guaranteed to all citizens and fund them through the National Health Fund. In the field of immunization, the Ministry of Health, in agreement with the State‐Region Conference, issues the Italian National Immunization Prevention Plan (Piano Nazionale di Prevenzione Vaccinale). This includes a set of vaccinations that are mandatory by law for all newborns. Mandatory childhood vaccinations are guaranteed free of charge for all Italians as well as foreign children who live in the country, and they are delivered in different doses up to 24 months of age. Regional health authorities can only set formal agreements about the offer of immunization not covered by the law, that is, they can increase the set of vaccinations offered. The access to the mandatory vaccines is thus uniform over the entire territory by law. This leads to a total sample of approximately 215 nonmissing observations. Data on regional broadband coverage are made available by the EUROSTAT; regional data on vaccines are provided by the Italian Ministry of Health. These include the percentage of the targeted population for all childhood vaccines (i.e., from 0 to 24 months). During the period considered, that is, 2006–2016, vaccines such as MMR, diphtheria‐pertussis‐tetanus (DTP), Polio (POL), and Hepatitis B (EpB) were mandatory by law, whereas *Haemophilus influenzae* type B (HIB) was highly recommended. These refer to coverage at 24 months for complete cycles of three doses of all vaccines but MMR, which is instead delivered in one dose.

We set up a DiD model as follows:

(1) $$Y_{\mathrm{rt}}=\beta_{1}\text{Post}+\beta_{2}\mathrm{BB}{\text{coverage}}_{r,t}+\beta_{12}\text{Post}\times {\text{BBcoverage}}_{r,t}+\delta X_{\mathrm{it}}+\mu_{r}+\lambda_{t}+\varepsilon_{\mathrm{rt}},$$

where *Y*_*rt*_ is the yearly regional immunization percentage rate for all types of child vaccines: MMR, DTP, HIB, POL, and EpB. *Post* is the indicator of the post‐2012 court decision period; whereas *BBcoverage* is our treatment intensity variable and measures the percentage of households that are connectable to broadband fixed and/or mobile connections. 2Following the EUROSTAT definition, broadband coverage at the local level is measured as “the percentage of households (with at least one member aged 16 to 74) that are connectable to an exchange that has been converted to support xDSL‐technology, to a cable network upgraded for internet traffic, or to other broadband technologies.” Note that this indicator refers to the connectable households and not to the households having a broadband subscription.
*X*_*it*_ is a vector of controls accounting for the socioeconomic development of the area, such as regional per‐capita disposable income and the share of university graduated individuals in the region. *μ*_*r*_ accounts for time‐invariant differences between regions; whereas, in order to preserve the parsimony due to the small sample size, time effects are taken into account through the inclusion of a linear trend in our model (*λ*_*t*_). Finally, *ε*_*rt*_ is the idiosyncratic error term.

To assess the robustness of our findings, we estimate two additional versions of Equation (1). First, we augment our DiD regression by adding linear region‐specific time trends. Second, in order to assess to what extent our estimates are affected by trends in regional socioeconomic development, we also estimate Equation (1) without the inclusion of socioeconomic controls (*X*_*it*_.) Lastly, following Bertrand, Duflo, and Mullainathan (2004), we perform randomization tests by estimating Equation (1) using a random selection of a set of different time periods and treatment intensities (Year × BBcoverage) and using these placebo treatments rather than the real one. We then perform a Monte Carlo simulation of these estimates with 2,000 repetitions in order to build a distribution of placebo treatment effects. This allows us to assess the credibility of the identification strategy and to check the robustness of our results to different assumptions about the structure of the error distribution.

## 3 RESULTS

In column 1 of Table 1, we report the DiD estimates of Equation (1) for all vaccines separately and for an overall measure of average immunization rates. We find a negative average treatment effect on all vaccines considered. Specifically, we find that a 10 percentage points increase in local broadband coverage led to a significant reduction of 1.23 percentage points in POL coverage, 1.14 in DTP coverage, 1.55 in EpB coverage, and 1.44 MMR coverage. To retrieve the relative size of the effect, we also present the percentage change with respect to the average immunization rates of each vaccine. This shows a similar reduction across the different vaccines (1.2–1.6%), with the highest effect found for the MMR and EpB. For the case of HIB, the spread of misinformation entailed a negative effect, although not statistically significant. In the period we considered, the HIB vaccine was only highly recommended, whereas all the others were compulsory. As misinformation was mainly surrounding compulsory vaccines, this could be a plausible explanation for the absence of a significant effect for this vaccine.

**Table 1:** Difference‐in‐Differences regression

	Baseline			Robustness checks			
	(1)			(2)		(3)	
Outcome	DiD	S.E.	%	DiD	S.E.	DiD	S.E.
POL	−0.123**	*0.044*	−1.3%	−0.101***	*0.034*	−0.129***	*0.039*
DTP	−0.114**	*0.043*	−1.2%	−0.101***	*0.032*	−0.119***	*0.040*
EpB	−0.155***	*0.043*	−1.6%	−0.131***	*0.038*	−0.161***	*0.039*
HIB	−0.073	*0.054*	−0.8%	−0.038	*0.088*	−0.076	*0.057*
MMR	−0.144**	*0.055*	−1.6%	−0.193***	*0.058*	−0.156***	*0.052*
ALL	−0.122***	*0.038*	−1.3%	−0.113***	*0.038*	−0.128***	*0.036*

*Note*. DiD coefficients of Fixed Effects estimates of Equation (1) according to several specifications. Column (1) represents the estimation of Equation (1). Column (2) includes a region‐specific time trend, whereas column (3) includes no control. Percentage change are calculated w.r.t. the average outcome rate in response to a 10% variation in the treatment intensity variable. Outcome variables defined as follows: vaccine coverage at 24 months for complete cycles (three doses) of Polio (POL), diphtheria‐pertussis‐tetanus (DTP), *Haemophilus influenzae* type B (HIB), Hepatitis B (EpB), and one dose of measles‐mumps‐rubella (MMR). ALL includes average immunization rates. Standard errors clustered at regional level in *italics*.

*** Statistically significant at 1%.

** Statistically significant at 5%.

* Statistically significant at 10%.

The magnitude of our results raises important public health implications. In fact, this reduction led immunization rates to reach below 95%, which is considered the herd immunization threshold. These results are also robust in magnitude to alternative specifications, that is, when augmenting Equation (1) by including region‐specific trends (column 2) and when excluding regional socioeconomic controls (column 3).

**Figure 2:**
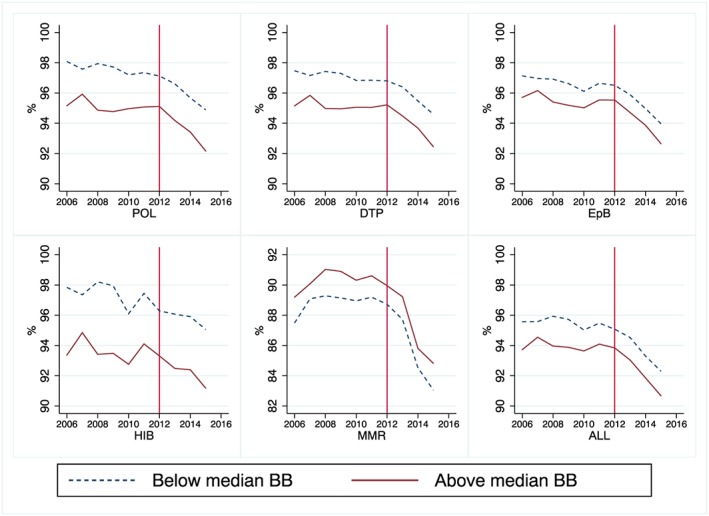
Trends in immunization rates across regions below and above the median of the treatment intensity variable. *Note*. The figure shows the precourt and postcourt ruling trends of immunization rates below (blue line) and above (red line) the time‐varying median of the regional broadband coverage. Outcome variables defined as follows: vaccine coverage at 24 months for complete cycles (three doses) of Polio (POL), diphtheria‐pertussis‐tetanus (DTP), *Haemophilus influenzae* type B (HIB), Hepatitis B (EpB), and one dose of measles‐mumps‐rubella (MMR). ALL includes average immunization rates. After the Court's decisions, the reduction in immunization rates was more marked for regions with a larger broadband coverage (red line) [Colour figure can be viewed at http://wileyonlinelibrary.com]

**Figure 3:**
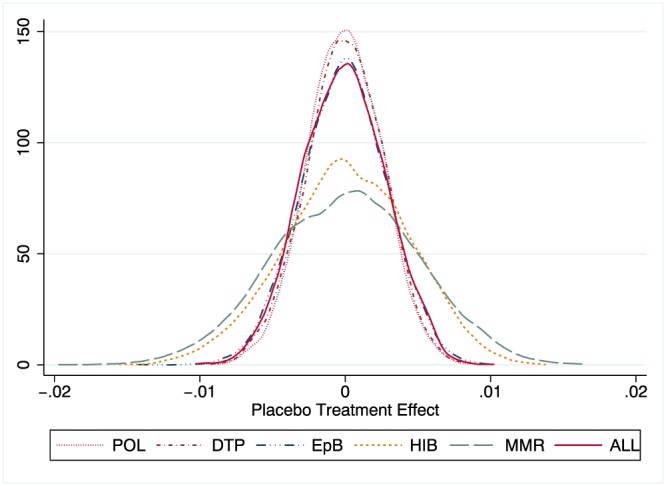
Placebo estimates. Kernel density distribution of 2000 placebo estimates for all types of vaccines [Colour figure can be viewed at http://wileyonlinelibrary.com]

In Figure 2, we perform a graphical inspection of the trends of immunization rates across regions below and above the time‐varying median of the treatment intensity variable. Visual inspection suggests that the common trend hypothesis can be credibly maintained. The figure also shows a visible drop in the immunization rates after the decision, and such a reduction is more marked for regions with a larger broadband coverage (red line). However, to reduce any residual concern about possible violations of common trend assumption, in Figure 3, we present the nonparametric distribution of placebo estimates. As the mean of the distribution is virtually zero, the estimator is unbiased. Moreover, all the average treatment effects we estimate in Table 1 fall in the very extreme left tail of this distribution. This increases the confidence that the effect we estimate is not obtained by chance and provides full support for our identification strategy.

## 4 CONCLUSIONS

Fake news and misinformation on social media are often blamed as the cause of the reduction in immunization rates worldwide. Recently, this has pressured policymakers, health authorities, and social media to seek regulatory interventions (see, e.g., Chiou & Tucker, 2018). Our paper aimed to provide causal evidence of the effects of fake news and misinformation on vaccine immunization rates. We exploited a quasi‐experiment that occurred in Italy when the Court of Rimini officially recognized a causal link between the MMR vaccine and autism and awarded vaccine‐injury compensation. After the decision, fake news and misinformation on vaccines went viral on the Internet. Building on growing literature studying the effects of the Internet on real‐life outcomes, we found that after the court's ruling in 2012, larger accessibility to nontraditional media (via broader broadband coverage) led to a reduction in child immunization rates. Interestingly, the negative and significant effect we found encompasses all vaccines and led immunization rates to reach below the critical threshold of 95%. Our results thus corroborate the thesis that misinformation is a dangerous cause of the vaccine hesitancy issue.
